# Weak supervision as an efficient approach for automated seizure detection in electroencephalography

**DOI:** 10.1038/s41746-020-0264-0

**Published:** 2020-04-20

**Authors:** Khaled Saab, Jared Dunnmon, Christopher Ré, Daniel Rubin, Christopher Lee-Messer

**Affiliations:** 1 Department of Electrical Engineering, Stanford University, Stanford, CA USA; 2 Department of Computer Science, Stanford University, Stanford, CA USA; 3 Department of Biomedical Data Science, Stanford University, Stanford, CA USA; 4 Department of Child Neurology, Stanford University, Stanford, CA USA

**Keywords:** Epilepsy, Epilepsy, Computer science

## Abstract

Automated seizure detection from electroencephalography (EEG) would improve the quality of patient care while reducing medical costs, but achieving reliably high performance across patients has proven difficult. Convolutional Neural Networks (CNNs) show promise in addressing this problem, but they are limited by a lack of large labeled training datasets. We propose using imperfect but plentiful archived annotations to train CNNs for automated, real-time EEG seizure detection across patients. While these weak annotations indicate possible seizures with precision scores as low as 0.37, they are commonly produced in large volumes within existing clinical workflows by a mixed group of technicians, fellows, students, and board-certified epileptologists. We find that CNNs trained using such weak annotations achieve Area Under the Receiver Operating Characteristic curve (AUROC) values of 0.93 and 0.94 for pediatric and adult seizure onset detection, respectively. Compared to currently deployed clinical software, our model provides a 31% increase (18 points) in F1-score for pediatric patients and a 17% increase (11 points) for adult patients. These results demonstrate that weak annotations, which are sustainably collected via existing clinical workflows, can be leveraged to produce clinically useful seizure detection models.

## Introduction

Seizures are an important cause of morbidity worldwide, with a lifetime prevalence of 5–10% in the global population. Seizures commonly result from an acute response to a brain insult such as trauma, stroke, or meningitis; such acute symptomatic seizures often cause injury secondary to their clinical manifestations and may exacerbate ongoing brain injury^1–5^. Seizures can also be chronic as in the case of epilepsy, which is defined as a disorder of the brain leading to an enduring predisposition to generate epileptic seizures (see Supplementary Note 1 for detailed definition)^6^. Epilepsy affects over 50 million individuals—nearly 1% of the global population—and leaves these patients with increased risks of injury, death, unemployment, depression, anxiety, permanent memory impairment, and many other psychiatric and psycho-social disorders^7–9^.

Monitoring at-risk patients for epileptic and acute symptomatic seizures is critical to making important therapeutic decisions. Because abnormal patterns of brain activity are an essential characteristic of seizures^7^, electroencephalography (EEG) analysis is the preferred method for seizure monitoring. EEG is a diagnostic test that detects epileptiform discharges (including electrographic seizures^7^) by monitoring voltage fluctuations caused by neural activity within the brain. While EEG-based seizure detection has yielded improved clinical results, these advances have come at the cost of massive physician burden. To perform seizure detection using EEG, an EEG reader must visually examine up to days of EEG signals to determine whether a pattern of abnormal electrical discharges indicative of a seizure has occurred.

Manual analysis of EEG data by board-certified EEG readers is extremely time-consuming and costly. A recent study of non-federal US hospitals concluded that continuous EEG monitoring accounted for an average of 5% of total hospital charges for monitored ICU patients^10^, and continuous EEG monitoring is rationed in practice because there are not an adequate number of interpreters to provide around-the-clock human monitoring for all patients who would benefit^11^. The increasingly large clinical burden of manual EEG analysis has motivated the recent development of automated algorithms for detecting seizures on EEG. Modern deep machine learning methods represent a particularly promising set of approaches for this task, as they have recently seen widespread success in medical domains including skin lesion classification from dermatoscopy^12^, automated interpretation of chest radiographs^13^, in-hospital mortality prediction from electronic health records^14^, and many others^15–21^. However, existing deep learning methods rely on the curation and continual maintenance of massive hand-labeled datasets, which has recently been identified as the major bottleneck in supervised medical machine learning^15^. As a result, many existing medical machine learning models are static with respect to changing patient populations, disease presentations, sensing hardware, and other variables. For instance, if a new task is required (e.g. detecting seizure sub-type), then a new labeled dataset must be curated. Further, recent analyses have shown that ensuring model generalization across patient populations with different characteristics remains a challenge, necessitating label curation and model retraining to deploy machine learning models to different demographics^22,23^. Practically, creating each new dataset could require physician-months or physician-years of labeling time, making repeated re-labeling campaigns a substantial diversion of resources. This problem is particularly salient for automated EEG monitoring, as achieving reliably high seizure detection performance across different patients even within the same population has proven difficult^24–28^.

**Fig. 1:**
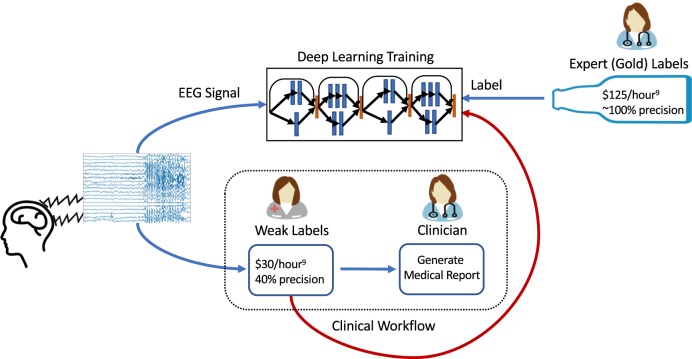
High-level view of integrating deep learning training within the clinical workflow. After EEG monitoring on patients, a mixed group annotates the signal before a clinician does the final assessment to generate a medical report. In our work, we evaluate using weak labels, which are fast and cheap to acquire within the clinical workflow, to train deep learning models for onset seizure detection. On the other hand, existing methods use expert-provided gold labels, which are costly and not a part of the clinical workflow.

In this work, we provide a strategy for training high-performance deep learning models for seizure onset detection that addresses these challenges. Instead of relying on expensive clinician-provided labels, we train deep learning models for seizure onset detection using noisier annotations that are imperfect, but inexpensive, and already being produced within existing clinical workflows. Specifically, when a patient undergoes EEG monitoring at a hospital, a mixed group of technicians, fellows, students, and board-certified epileptologists preliminarily analyze the EEG signal to provide initial annotations of seizure times, which a board-certified clinician eventually uses as the starting point for their own analysis^11^. We refer to these initial annotations as weak annotations because of the specific circumstances in which they are produced. Such weak annotations are meant to be helpful for clinicians, but they are not required to be complete, and thus produce only sparse labeling of seizures in any given signal. In current clinical practice, experienced technicians provide the majority of these initial weak annotations. These personnel are specifically instructed to mark suspicious EEG segments while erring on the side of high sensitivity in ambiguous cases; this procedure leads to annotations with an inherently high false-positive rate. However, because each EEG signal may contain a large number of distinct seizures, these annotators usually mark only a subset of the seizures in a given signal, meaning that overall annotation recall is also modest. Further, a subset of seizure annotations comes from medical students and fellows with varying levels of experience, resulting in annotations that are generally less reliable than those provided by the technicians. Each of these human annotators may also have used other data modalities, such as patient videos, to inform their annotation. Thus, a seizure may be annotated even though nothing indicative of a seizure is present in the EEG signal. To estimate the quality of these weak annotations as labels for a machine learning model, a board-certified clinician (Dr. Christopher Lee-Messer, or CLM) annotated every seizure in 30 pediatric EEG signals that included 32 seizures, and 82 adult EEG signals that included 91 seizures, sourced from the dataset used in this work. The weak annotations in those signals had an overall precision of 0.37 and recall of 0.45. While most previous studies^24–28^ use clinician-generated labels for training, we propose to directly use these weak annotations for model supervision instead (Fig. 1). This approach creates a sustainable workflow for training deep learning algorithms for seizure detection that could be continually updated and improved with no additional resources with respect to current practice. Accurate automated seizure detection tools trained using weak annotations could reduce existing clinician and technician burden while simultaneously improving quality of care, lowering costs, and expanding access to EEG monitoring services.

In this work, we present a deep learning model for the detection of seizure onset across different patients that is trained on a massive, weakly labeled dataset of scalp EEGs. This approach formally falls within the scope of weak supervision techniques, which include data programming^29^, distant supervision^30^, and crowdsourcing^31^. We consider training labels drawn only from the weak annotations included with each EEG signal archived by a pediatric and an adult hospital at our institution—that is, we use no data hand-labeled by expert clinicians for model training. In total, our dataset consists of 136,014 EEG signals coming from 12,430 patients. For perspective, to obtain clinician-provided hand-labels for the number of signals we consider in this study—which cover 12 years of clinical practice at a major center—we conservatively estimate that it would require over 25,000 person-hours and $3 million dollars in clinician support (136,014 EEG signals, $125/hour for the clinician rate^11^, and a median EEG read time of 12.5 min^32^). Since clinicians usually read EEG signals in 10–15 s-long pages, we observed that some seizures may be detected in the scope of a single page, while other seizures require viewing multiple pages after seizure onset for accurate detection. We consider these different time scales by building models for 12-s clips to simulate fast detection (i.e. single-page) scenarios, and 60-s clips to simulate slow detection (i.e. multi-page) scenarios (see Supplementary Fig. 1 for examples). In each case, we evaluate the effectiveness of using weak annotations for model supervision. We use only the most common 10–20 subset of EEG leads to ensure that our model is applicable to as many patients as possible. Our fast detection models achieve an average Area Under the Receiver Operating Characteristic Curve (AUROC) of 0.91 on pediatric patients, while attaining a lower average (AUROC) of 0.82 on adult patients. On the other hand, our slow-detection models provide improved performance levels of 0.93 AUROC on pediatrics and 0.94 AUROC on adults. We also compare our models with traditional machine learning approaches for seizure detection, with models trained on a publicly available EEG database, and with Persyst, a leading provider of clinically deployed software for automatically detecting seizures from EEG^33^. Our models consistently outperform those trained using either traditional machine learning approaches on our dataset or the same model architecture on a publicly available EEG database. Further, our approach achieves higher performance than Persyst by 18 and 11 F1-points for pediatric and adult populations respectively, which suggests immediate clinical utility. In the following sections, we present related work, describe our experimental setup, evaluate the performance of our models, assess our results in the context of publicly available datasets, and analyze success and failure modes of the learned models from a clinical perspective.

Researchers have previously tackled the problem of detecting the transition from the preictal period, which precedes seizure to the ictal period that is characterized by seizure. This is a challenging task because the transition between the preictal and ictal periods is often subtle^24^. Further, because seizures have patient-specific characteristics, many detection algorithms require training on each individual patient^24–28^. Bandarabadi et al.^24^ propose seizure detection by using amplitude distribution histograms of features extracted from EEG recordings, such as spectral power features from different frequency bands. While the authors propose a novel yet simple statistical method, model parameters need to be tuned by training on previous seizures for each patient. Moreover, their dataset consists of 2693 h of EEG recordings from over 18 patients and was annotated through visual inspection by epileptologists, along with analysis of video recordings of each patient. Ozdemir et al.^25^ use intracranial EEG signals to develop an automated seizure detection system, which extracts features using a Hilbert-Huang transform and uses a Bayesian network for classification. The authors use the Freiburg EEG database and had a certified epileptologist visually inspect 577 h of iEEG recordings to provide annotations at the electrode-level. Ozdemir et al. obtained impressive results of 96.55% sensitivity and 0.21 false alarms per hour; however, their model is patient-specific and requires intracranial EEG recordings, which necessitate invasive surgery and are not relevant for the large patient population with scalp-only EEGs. Persyst is the trade name of a common, clinically deployed commercial seizure auto-detection software that is based on automated analysis of raw EEG signals and various spectrograms using a combination of rule-based approaches and neural networks^33^. In practice, rule-based systems such as those described above are notably brittle with respect to new sensors, data containing common artifacts, and generalizing to previously unseen patients. We evaluate some of these same potential drawbacks in the context of our deep learning approach in the Results section, and use Persyst as a baseline against which we evaluate our models.

While many of the previously mentioned works rely on hand-engineered features generated from the EEG recordings, deep learning models have gained wide popularity for their ability to automatically learn features from the input data that achieve state-of-the-art performance on many medical analysis tasks. However, a hidden cost of these deep learning models is that they incorporate large numbers of trainable parameters, and therefore they typically require massive labeled training datasets to achieve high performance. Ullah et al.^34^ proposed a system composed of an ensemble of pyramidal one-dimensional Convolutional Neural Networks (CNNs). They used the University of Bonn dataset, which consists of EEG signals from 10 patients that were visually inspected by expert neurologists. They also use two data augmentation schemes and report a test accuracy of 99%. However, these authors train and test on the same patients (five normal and five epileptic patients). Acharya et al.^35^ used the same dataset as Ullah et al., and implemented a 12-layer CNN to detect normal, preictal, and ictal (seizure) classes from EEG signals and achieve 88.67% accuracy. More relevant to the present work, Temple University Hospital (TUH) recently released the world’s largest publicly available EEG dataset^36^ containing the time of occurrence and type of 2,012 seizures (v. 1.4). Asif et al.^37^ recently used this dataset to achieve a weighted F1-score up to 0.90 for seizure detection using a hand-engineered preprocessing pipeline to extract features coupled with a densely connected CNN (Dense-CNN). While the TUH dataset does offer the opportunity to train deep learning models using a large corpus of EEG signals, its curation required trained students to hand-label 2997 EEG signals, which is not sustainable for rapidly curating new datasets to keep pace with evolving patient populations and sensing technologies. We address this challenge by evaluating the performance of deep learning models using weak annotations instead, which allows us to use a dataset with over 40 times more EEG signals than available from TUH. Since these annotations are a natural part of the existing clinical workflow, our approach represents a sustainable pipeline for developing automated seizure detection systems.

## Results

### Datasets

**Fig. 2:**
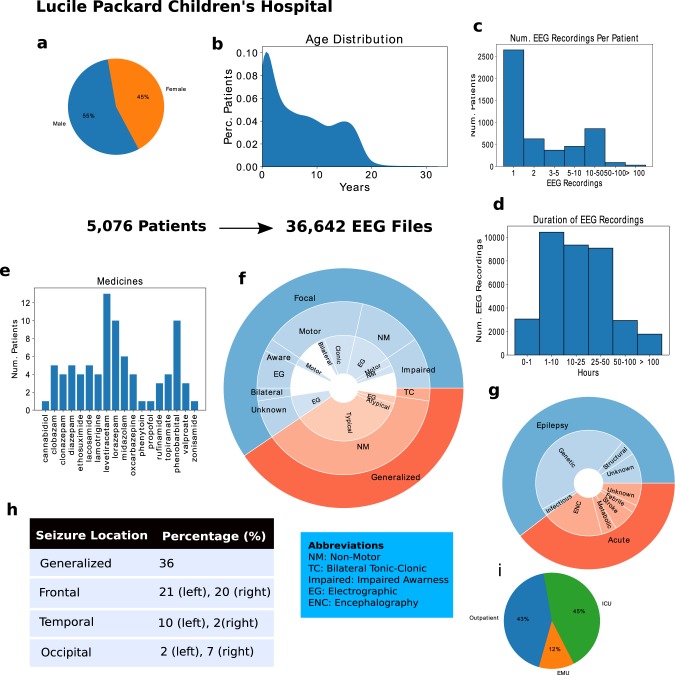
Data description for Lucile Packard Children’s Hospital (LPCH). **a** the gender distribution, **b** the age distribution, **c** the number of EEG recordings per patient, **d** the durations of EEG recordings in hours, **e** a histogram of the medicine types, **f** a hierarchical pie graph describing the different seizure types, **g** a hierarchical pie graph describing etiology types, **h** the percentages of EEG signals collected in different locations, and **i** the distribution of seizure locations. Subfigures **a**–**d** are statistics on the entire dataset, while **e**–**i** are on the held-out test set that included 52 seizures.

We approach the seizure onset detection problem as a classification task over both 12-s (fast detection) and 60-s (slow detection) EEG clips. After undersampling the plentiful negative examples to obtain a balanced dataset, our pediatric seizure onset detection dataset contains 25,386 class-balanced EEG clips for training, and 498 additional EEG clips with gold-standard labels provided by a board-certified EEG reader (CLM). For the adult dataset, we have 32,596 class-balanced EEG clips for training, and 480 additional EEG clips with gold-standard labels. For each population, the gold-labeled data are divided equally into a development set, used to tune hyperparameters during training, and a held-out test set, used for final evaluation of our trained model. Clinical details of our datasets are summarized in Fig. 2 for the pediatric dataset and Supplementary Fig. 2 for the adult dataset. Additional detail on model architecture, data preprocessing, and model training procedures can be found in the Methods section.

### Evaluation

We first validate that deep learning performs better than traditional machine learning approaches for the task of cross-patient seizure onset detection. Traditional machine learning approaches to discriminate preictal EEG segments from ictal ones consist of training classifiers such as Logistic Regression (LR), Support Vector Machines (SVM), and Random Forests (RF) using hand-engineered time-frequency domain features^38,39^. We follow procedures in Schiratti et al.^38^ to train LR and RF classifiers for the task of seizure onset detection given 12-s or 60-s EEG clips over the same datasets described above. We use the same hyperparameters for RF training as Schiratti et al.^38^, which is 100 estimators and a maximum depth of 4. A variety of commonly used features^39^ were extracted from each EEG clip to support the analysis; these include time domain features such mean, variance, skewness, kurtosis, total signal area, peak-to-peak, number of zero-crossings, and decorrelation times; frequency domain features such as total energy spectrum, energy percentage across fundamental rhythmic bands (extracted using the Discrete Fourier Transform), and the coefficients from the Discrete Wavelet Transform; brain connectivity features such as the maximal absolute cross-correlation value to measure similarity between electrodes; and local and global electrode graph measures^38–41^.

Second, we validate that we achieve clinically useful performance by comparing our model to Persyst Version 13 (Persyst-13), a state-of-the-art commercial EEG software package used for automated detection of seizures and spikes^33^, on the same evaluation set.

Third, to show that training our deep learning model over a large, weakly annotated dataset is advantageous, we compare the performance of the same CNN architecture trained over (1) the large weakly annotated dataset, (2) a small gold-labeled dataset (i.e., our hand-labeled development set), and (3) a large, publicly available hand-labeled EEG dataset (TUH). We also show how the performance of the model scales as we increase the amount of weakly labeled training data.

Fourth, we evaluate how well the trained models can generalize across different patient populations and hospitals. To do this, we evaluate the model trained using adult EEGs on the pediatric test set (and vice-versa), and assess our models trained on large weakly labeled datasets against the TUH dataset (and vice-versa).

Fifth, we evaluate the success and failure modes of our fast detection model trained over the weak annotations for the pediatric population. We perform this error analysis by having a trained neurologist examine how the model prediction changes over time for complete EEG signals within our held-out test set. In addition to analyzing model predictions, we also extract heatmaps for each clip that indicate which time-intervals and EEG leads have the most influence on a given model prediction. Each heatmap is calculated by occluding (i.e., replacing with zeros) one-second segments across each 12-s EEG clip for each of the 19 channels, and observing the percent change in the model’s prediction. These heatmaps represent the degree to which each one-second segment of each channel contributes to a positive model prediction.

### Comparison to baseline methods

Since the output of Persyst is a binary indication of seizure without parameters to adjust false-positive or true-positive rates, AUROC is not a meaningful metric. Therefore, we compare the F1-scores and false-positive rates (FPRs) on our test set for the two classical machine learning classifiers (Logistic Regression and Random Forest), Persyst, and our CNN model trained over either a small gold-labeled training set or the large weakly annotated training set in Table 1 (refer to Supplementary Tables 1 and 2 for confidence intervals). We observe that our CNN model trained on weak labels substantially outperforms all baselines on both adult and pediatric datasets with respect to the F1-score metric.

**Table 1:** Comparison of baseline models to the weakly supervised CNN using the F1-score and FPR metrics.

Performance comparison of baseline models to the weakly supervised model				
	Pediatric (F1-score, FPR)		Adult (F1-score, FPR)	
	12-s	60-s	12-s	60-s
Logistic regression	0.25, 0.36	0.37, 0.50	0.24, 0.56	0.38, 0.55
Random forest	0.38, 0.19	0.56, 0.52	0.37, 0.61	0.58. 0.52
Persyst-13	0.07, 0.015	0.61, 0.10	0, 0	0.65, 0.054
Dense-CNN (small gold-standard set)	0.29, 0.39	0.60, 0.20	0.28, 0.47	0.41, 0.16
Dense-CNN (weak annotations)	0.67, 0.13	0.77, 0.10	0.49, 0.14	0.76, 0.079

The F1-score of our weakly supervised CNN outperforms the baselines by substantial margins. The small gold-standard training dataset consisted of 241 clips for pediatrics and 246 clips for adults. The weakly annotated training dataset consisted of 25,386 clips for pediatrics and 32,596 clips for adults.

### Model performance and scaling analysis

**Fig. 3:**
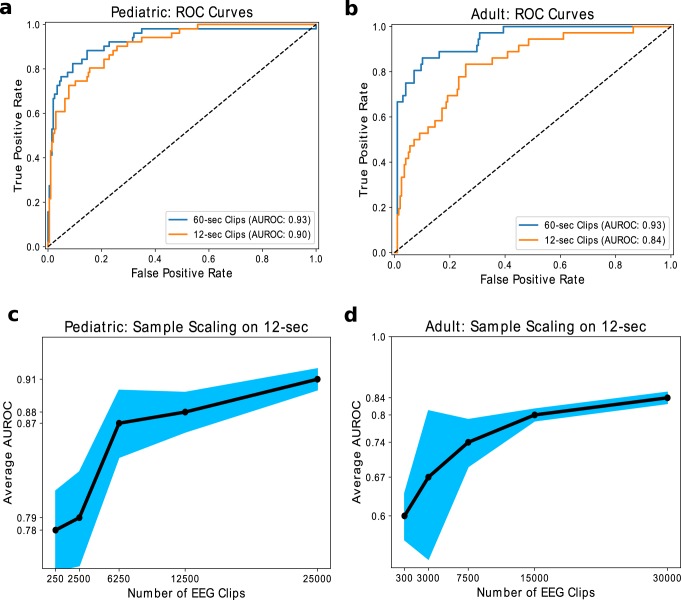
ROC curves and sample scaling results. We plot **a** median ROC curves when evaluating our model trained on 25,386 weak labels over 60-s and 12-s EEG clips for pediatric patients and **b** median ROC curves when evaluating our model trained on 32,596 weak labels over 60-s and 12-s EEG clips for adult patients. We also show model performance versus the number of weak labels used for training over pediatric patients (**c**) and adult patients (**d**) over the 12-s EEG clips. Error bars represent 95% confidence intervals from five training runs with different random seeds.

In Fig. 3, we compare how well models perform on adult and pediatric test sets when using a large weakly annotated training set over both 12-s and 60-s clips. For pediatric patients, we achieve an average AUROC of 0.91 over five trials when we train our model for 12-s EEG clip seizure detection, compared to an average AUROC of 0.93 when trained for 60-s EEG clips (Fig. 3a). For adult patients, we achieve an average AUROC of 0.82 when we train over 12-s EEG clips, compared to an average AUROC of 0.94 when trained over the 60-s EEG clips (Fig. 3b). We also trained our fast detection models with varying training set sizes to observe how model performance scales with sample size. In Fig. 3c, d, we present the average AUROC on the test set trained over five random seeds. We not only observe rapid performance improvement with additional examples, but also demonstrate a reduction in model variance across training runs with different random seeds. Thus, our scaling results suggest that the performance of machine learning models supervised by imperfect weak annotations continues to improve as more weakly labeled data are added, which is consistent with the results of recent studies of weak supervision techniques^42^.

### Performance on different patient populations

**Fig. 4:**
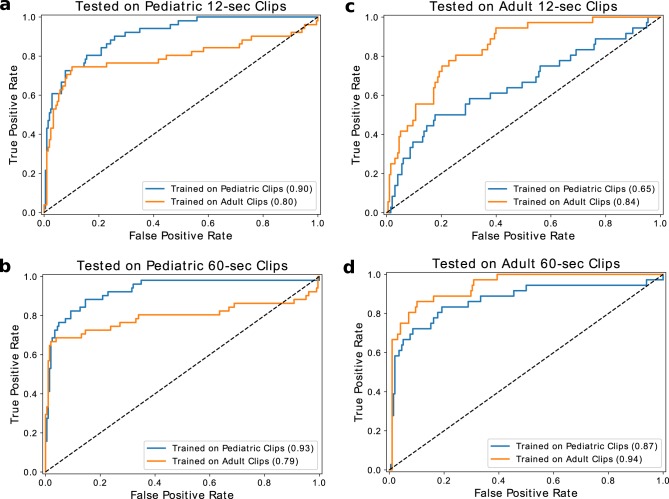
Generalization performance across pediatric and adult populations. We demonstrate the difference in performance when the model is trained on the two datasets and tested on the pediatric clips (**a**, **b**), and the adult clips (**c**, **d**). The orange lines indicate models trained on the pediatric population, while the blue lines indicate models trained on the adult population.

An increasing concern in medical machine learning is how well a trained model is able to perform on heterogeneous patient populations at test time. In our case, we are interested in how our models, which have either only seen pediatric or adult patient data during training, would perform when evaluated against the other age group. As seen in Fig. 4, there was a substantial decrease of 26 AUROC points in performance for the median model trained on pediatric patients that was tested on adult patients in the fast detection regime. On the other hand, there was a decrease of only 4 AUROC points in performance for the median model trained on adult patients and tested on pediatric patients. Interestingly, these generalization trends change in the slow-detection regime, where we observe a 7 AUROC point decrease if the pediatric model is tested on adults versus a 14 AUROC point decrease if the adult model is tested on pediatric patients. These results suggest that adult and pediatric patients are different enough that age-naïve models should not be expected to reliably generalize from one population to the other.

**Fig. 5:**
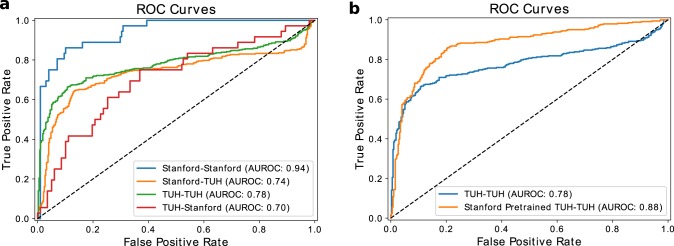
Generalization and transfer learning results across institutions. **a** We train models using both Stanford and TUH datasets, and evaluate on the test set from each institution. We show median ROC curves obtained from 5 runs of each model. X–Y stands for trained on X and evaluated on Y. **b** We fine-tune our model pre-trained on the large weakly labeled Stanford dataset using the TUH dataset. We compare the median ROC curves with and without pre-training.

We also evaluate model performance on EEG data coming from a different hospital, as distributional shifts in the EEG data resultant from changes in the patient population, the measurement hardware, or perhaps even the protocols followed by clinical staff could cause performance degradation. To measure these effects, we evaluated our model trained on the 60-s Stanford dataset against 60-s seizure onset clips from the TUH test set. We also evaluated the model trained on TUH seizure onset clips against the Stanford test set. As seen in Fig. 5a, our Stanford model performed 4 points AUROC worse than the TUH model when evaluated against the TUH test set, while our TUH model performed 24 AUROC points worse than the Stanford model when evaluated against the Stanford test set. These observations support the idea that deep learning models do not necessarily transfer directly from one institution to another, and that the ability to train weakly supervised models on an institution-by-institution basis may provide value in practice. Further, we find that the model trained on TUH and tested on TUH, using the same model architecture and training procedures, performed 16 AUROC points worse that the Stanford model tested on Stanford. These results further support the conclusion that large, weakly labeled datasets may have practical advantages over smaller gold-labeled datasets.

We also assess whether the Stanford data can improve model performance on TUH via transfer learning. Specifically, because the Stanford model was trained on a larger dataset covering a wide range of seizures, we might expect improvements when performing transfer learning from Stanford to TUH. To test this hypothesis, we fine-tune the last two fully connected layers of the Stanford model using the TUH dataset, with the rest of the model parameters frozen. As shown in Fig. 5b, we observe a substantial improvement of 10 points AUROC when fine-tuning our Stanford-trained model on the TUH dataset with respect to training a model on the smaller TUH dataset. These results suggest that our weakly supervised models have learned a representation of the data that is broadly helpful for identifying seizure onset, as this representation is useful in seizure onset detection on data from a different hospital.

### Error analysis

**Fig. 6:**
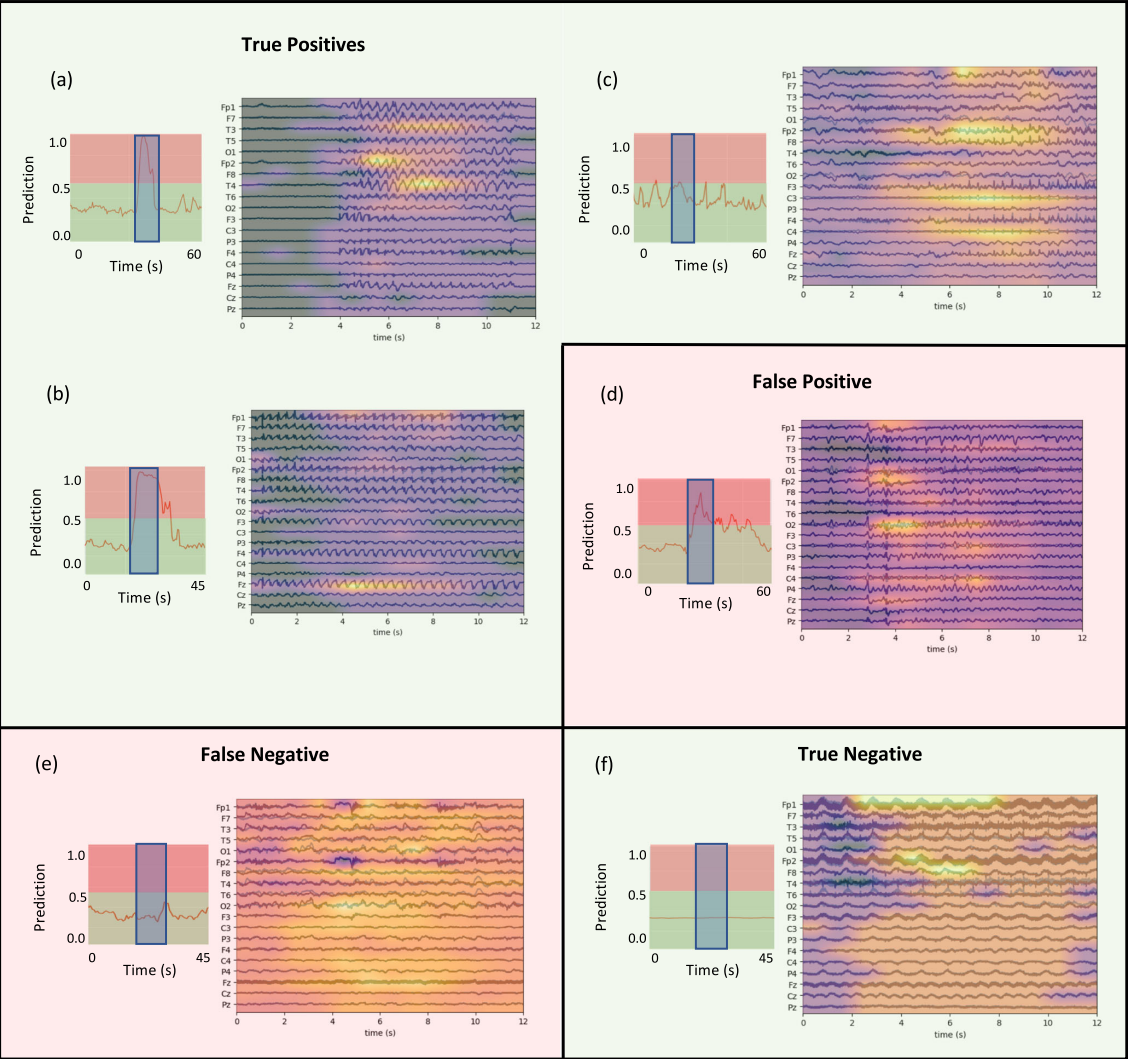
Representative cases for true-positive, false-positive, false-negative, and true-negative. Green background represents successful cases, while the red background represents failure cases. The EEG clips are shown and overlaid with an occlusion map. To the left of the clip is a plot indicating the model prediction values in the vicinity of the clip, where the clip is highlighted by the blue rectangles. Note that occlusion map values are normalized within each image, meaning that values should not be compared across images.

While we train deep learning models for both fast and slow seizure onset detection over pediatric and adult populations, we focus our detailed clinical error analysis on the pediatric fast detection model, which achieves high levels of performance (0.91 AUROC). The fast detection task is challenging even for clinicians, who generally have access to additional information including baseline EEG activity contained in the remainder of the EEG signal, video data of the patient, medical imaging studies, previous clinical reports, and other detailed patient history to guide their EEG interpretation. For our error analysis, a fellowship-trained EEG reader identified representative clips for true-positive, false-positive, false-negative, and true-negative examples. In Fig. 6, we display each clip, overlaid with a heatmap indicating the parts of the clip that most influenced model prediction, along with model prediction values before, during, and after the clip. In Supplementary Figs 3 and 4, we compare these heatmaps to those generated on the exact same samples using the model trained on the small, gold-labeled dataset and show that the weakly supervised model tends to localize salient seizure activity more sharply.

Figure 6a shows an example in which a frontally-predominant generalized seizure is correctly detected by our model. Of note, the heatmap demonstrates that the model is sensitive to higher amplitude spike and wave regions in frontal and temporal regions with less sensitivity to the waveforms in lower-amplitude channels. Also, it is not heavily influenced by the lower-amplitude background before and after this brief seizure. The fact that the model is most influenced by channels that exhibit a consistent pattern (i.e., Fp1, Fp2, T3, T4) suggests that our pediatric fast detection model uses common mechanisms to detect focal and generalized spike-wave seizures.

The true-positive example in Fig. 6b indicates a similar model response to a generalized spike-wave seizure. Further, it illustrates another potentially useful phenomenon: the model tends to output high abnormal probabilities over the duration of most seizures. While this behavior is not necessarily unexpected given that the weak technician annotations tend to be—but are not always—near the beginning of a seizure, it suggests that models weakly supervised with clinician annotations could nonetheless be useful for measuring seizure duration in addition to performing seizure detection.

The seizure shown in Fig. 6c illustrates model response to a relatively subtle focal seizure involving fast spiking activity which evolves with predominant activity in the right frontal central regions, but with a bilateral field. In this case, the model prediction appears most sensitive to activity in the frontal and central regions. Encouragingly, the model is able to correctly identify at least some focal seizures in addition to more obvious generalized seizures.

The false-positive case of Fig. 6d is a good example of the sort of rhythmic activity which can fool the model, as its prediction of seizure probability is consistently in the 0.7–0.9 range. Though this activity was ultimately judged to be non-ictal by the clinician reader due to lack of clinical correlate, long-term evolution, and similarity to interictal activity, over the short term it certainly displays some ictal characteristics. For instance, if we look closely at the occlusion map, the highly rhythmic, evolving activity starting 3 s from the beginning of the clip is most salient in the model prediction. In a different long-term context, an epileptologist could well interpret these same features as being indicative of seizure.

The false-negative of Fig. 6e represents a focal seizure that starts with rhythmic, slow activity in the frontal regions. While 25 s later the pattern evolves to clearly have rhythmic sharp activity, which the model classifies as a seizure, the early activity is more subtle. It is likely that this case is challenging for the model used in this work because unlike the human marking the EEGs, the clip-based model has no ability to resolve ambiguities in early patterns using information from later time points in the signal. In addition, rhythmic slowing can not only represent seizure, but also overlaps with non-ictal patterns such as frontal interictal rhythmic delta (FIRDA). Such issues could potentially be addressed in future refinements to the model architecture.

Figure 6f shows an example of an EEG with large amounts of non-evolving, rhythmic artifact and 60 Hz AC line noise. The model correctly classifies this as non-ictal. The occlusion analysis indicates that it weights the channels roughly equally in making this overall determination. This is an important example because this EEG pattern is likely to trigger false detection using many of the baseline methods. In fact, we found that Persyst indeed incorrectly classified it as a seizure. This true-negative case indicates the substantial practical utility of a large dataset which includes multiple examples of artifacts that can often fool both inexperienced EEG readers and simpler models. Encouragingly, the true-negative of Fig. 6f also shows few oscillations in the predicted value as the CNN detector is slid over the signal. Further, the relatively uniform signal in the occlusion map indicates that the model is not incorrectly identifying certain areas in this clip as more seizure-like than others. Overall, close analysis of representative success and failure modes indicates that the model has learned features that are indeed meaningful for clinical interpretation.

## Discussion

The results of Table 1 clearly indicate that the CNN model provides the best seizure onset detection observed in this study for both 12-s and 60-s EEG clips. Indeed, the fact that we observe an 18 F1-point advantage in pediatric populations over systems that are currently in clinical use suggests that our model could have substantial clinical utility. One reason why the model presented here outperforms the baselines so substantially may be the difference in datasets and tasks considered in the literature when these baselines were developed. For example, Ullah et al.^34^ and Schiratti et al.^38^ train and test on the same patients using a much smaller cohort (e.g., 10 patients) rather than on a large cohort with different patients in the train and test sets. To determine whether this large difference in performance is at least partially due to the challenge of cross-patient seizure detection, we trained the same Random Forest model as in Table 1 on a single patient, and tested its ability to predict a seizure for that same patient. In this experiment, the Random Forest model achieved an F1-score of 0.77 when evaluated on the same patient whose data it was trained on, whereas for the cross-patient task it achieved an F1-score of only 0.37. These results, along with the error analysis presented in Fig. 6, suggest that modern deep learning architectures have the ability to learn features that are more suitable for cross-patient seizure detection than hand-engineered alternatives.

Comparing Fig. 3a with Fig. 3b, we also see that the model trained over the pediatric population achieves significantly higher performance, by 9 AUROC points, than the model trained over the adult population for 12-s clips. Possible reasons for this gap could be (1) the higher prevalence of focal seizure with evolving slowing over long periods in adults versus more generalized seizures in pediatrics, which are easier to detect over short periods, (2) signal differences (e.g., pediatric heads are smaller), and (3) differences in annotation procedure (e.g., mixture of expertise) between pediatric and adult hospitals.

To inform an analysis of translational potential, it is useful to assess specific operating points in addition to aggregate AUROC values. For instance, a potential operating point in pediatric patients for fast detection could be the threshold that achieves a true-positive rate (TPR) of 90% with a false-positive rate (FPR) of 25%, or a TPR of 92% and FPR of 24% for slow detection. This translates to missing one-tenth of the clips with seizure in a signal with a quarter of the predicted seizure clips being false-positives. While such percentages are not clinically acceptable for final decision making, a model that captures 90% of the seizure clips in a signal can be of great use to expedite the physician analysis process. On the other hand, a potential operating point for the model trained over the adult population for fast detection could be the threshold that achieves a true-positive rate of 92%, with a false-positive rate of 35%. For a similar false-positive rate of 25% as the pediatric model, the true-positive rate would drop down to 64%, which is undesirable. Therefore, assuming we chose the first operating point, the physician would need to accept 10% more false-positives for the adult population. However, for slow detection on adults, we would be able to operate at a TPR of 92% and FPR of 24%, the same as that of pediatrics. While our trained models perform well on our held-out test sets, a current limitation is that our dataset consists of EEG signals coming from a single institution. Therefore, as shown in Fig. 5, such performance levels may not hold for EEG signals coming from unseen institutions, perhaps due to variability in sensing equipment or demographic patient distributions. However, each institution could use their own weak annotations to train or fine-tune models optimized for their populations; in practice, this would require a hospital to either maintain in-house expertise for model training or supply weakly annotated data to an external vendor. As is the case with currently deployed systems such as Persyst, robust procedures for pre-deployment testing and post-deployment monitoring will be critical to ensure that such models provide value to both clinicians and patients.

An important result shown in Fig. 4 is that models trained on adult or pediatric patient populations do not necessarily generalize well to one another. Encouragingly, Fig. 5 demonstrates that transfer learning from large, weakly supervised datasets may help to improve model performance on new populations. Compelling directions for further mitigating such generalizability challenges in future work include multitask learning, data augmentation, and recent statistical techniques for weak supervision. In multitask learning, for instance, a model could be designed to also predict the age of a patient, which would enable the model to learn age-related features that are useful for seizure detection. In data augmentation, one might purposely add a wide range of age-related artifacts, which would enable the model to perform well on data from a broader population. Recent statistical approaches to weak supervision, such as the Snorkel framework^42^, could be used for model training either by estimating the accuracies of different annotation sources (i.e., different technicians) or by integrating additional information contained in free text EEG reports^43^. Moreover, if annotators in the clinical workflow were to further standardize their annotations, we may also see an increase in both the quality of supervision signal and the number of useful tasks that could be supervised without substantially increasing workload. Future research on automated seizure detection should also take the demography-related performance variations observed for the fast detection task in this work into account.

In this work, we provide evidence that deep learning models for seizure onset detection can outperform currently deployed clinical techniques. Our main contribution is the development of such models using weak annotations, a noisier source of supervision, instead of manual labels provided by clinicians. The supervision approach we propose yields high levels of performance compared to systems currently deployed for clinical use, allows for rapid training of institution-specific models, supports rapid retraining for model maintenance in the context of evolving patient demographics, and yields representations that are useful for transfer learning across institutions. Since weak annotations are already collected in the clinical workflow (i.e., are essentially available for free), our work shows that it is possible to sustain the training of data-hungry deep-learning models over EEG data that perform well across patients. While challenges remain in determining the optimal way to deploy models that vary in performance across different patient subpopulations, modern methods such as data augmentation, transfer learning, and multitask learning may offer a way forward. In the future, we will experiment with using models such as graph neural networks (which are naturally suited to the EEG domain), integrate additional temporal information using recurrent neural networks, assess our ability to build models that classify seizure subtypes in addition to performing detection, analyze model performance by demographic in a more fine-grained manner, and assess generalizability of our approach across multiple institutions.

## Methods

### Datasets

Patient data collection for this study was approved by the Stanford University Institutional Research Board under protocol IRB-37949. A waiver of consent was granted based upon the findings of minimal risks to patient welfare or rights and the impracticality of obtaining consent retrospectively for thousands of patients in studies occurring over many years. We collected two large scalp EEG datasets from our institution to evaluate how deep learning models perform when supervised with archived weak labels. The first dataset we collected contains 5,076 unique pediatric patients with 36,644 EEG signals from the Lucile Packard Children’s Hospital, where each EEG signal is an average of 43.8 h in duration and was annotated by a mixed group of technicians, fellows, students, and boarded epileptologists in a freeform manner, which included the annotation of possible seizures via stereotyped text such as seizure inserted at a specific time in the record. While these annotation times are not gold-standard labels, they are already provided as part of the standard clinical workflow. Our second dataset has 7,363 unique adult patients with 99,721 EEG signals from Stanford Hospital, where each EEG signal is an average of 22.6 h in duration and also contains weak annotations of seizure times. We observed a strong—but not absolute—tendency for these annotations to come near the onset of seizures.

The EEG signals in these datasets cover the past 12 years of clinical practice at our institution; as such, they encompass all seizure types encountered at a Level 4 pediatric and adult epilepsy center with associated hospitals that treat high-acuity patients in neonatal, pediatric and adult intensive care units.

For both adult and pediatric populations, we curated development and test datasets where seizure onset times were determined by a fellowship-trained EEG reader with board certification in epilepsy (CLM). CLM further analyzed the medical reports in every EEG signal used in each test set to characterize the seizure types, seizure locations, recording locations, seizure etiologies, and medications in use. Seizure types and etiologies were determined based upon the latest ILAE guidelines (see Supplementary Note 2 for details)^44^. Additionally, we calculated statistics on each weakly labeled training dataset, such as the distributions of gender, age, number of EEG signals per patient, and duration of the EEG signals (Fig. 2, Supplementary Fig. 2).

To ensure consistency across EEG signals, each of which could have a unique sensor alignment, we use only signals from the 19 electrodes in the standard 10–20 International EEG configuration, which form a subset of the electrodes deployed to every patient at our institution. This excludes premature infants and patients with small heads or injuries which prevent full deployment of these 19 electrodes. Infants whose heads could accommodate the full 19 electrode montage were included. Voltage readings from each channel are sampled at 200 Hz.

We transform the seizure onset detection problem into a clip-level classification problem over 12-s and 60-s clips. It is worth noting that clip length could be further optimized via hyperparameter search, but this was not a focus of our study. Our models either map an input, $x \in {\Bbb R}^{2400 \times 19}$ (12-s clips are sampled at 200 Hz over 19 leads) or $x \in {\Bbb R}^{12000 \times 19}$ (for 60-s clips), to a scalar output *y* indicating the probability of seizure onset in that clip. For negative labels, we sample random EEG clips from signals that do not have any technician-provided annotation times. Since there are many more cases without seizures than with seizures (roughly an 80–20 class balance percentage), we balance the training set by undersampling the negative examples. We had EEG readers label for 12 h and ended up with ~100 positive examples (80 and 98, respectively) for adult and pediatric populations. We then balanced the test set to have 80–20 negative-positive balance, to reflect the relative rarity of seizures. For the pediatric dataset, we are left with 25,386 class-balanced EEG clips for training, and 498 EEG clips with gold-standard labels. For the adult dataset, we are left with 32,596 class-balanced EEG clips for training, and 480 EEG clips with gold-standard labels. The total combined EEG signals for pediatrics and adults used for training is 72,628.

For models trained on the publicly available TUH dataset, we used version 1.4 of the dataset, which includes a training set and an evaluation set. The training set is composed of 1984 EEG signals coming from 264 patients, and contained a total of 1,327 seizure clips. In the evaluation set, which was split evenly between validation and test, there are 1,013 EEG files from 50 patients, which have a total of 685 seizure clips. To evaluate our Stanford model on the TUH dataset and vice-versa, we ensured that the input data to the models were the same with respect to the EEG leads used and the samples per EEG lead. Since the Stanford data were sampled at 200 Hz and TUH signals were sampled at varying frequencies (256 Hz, 512 Hz, etc.), we resampled all TUH signals to 200 Hz using resample function in the resampy python library with the Kaiser best windowing technique. We did not de-reference signals from TUH.

### Performance metrics

To assess how well the different models in this work can detect seizures, we computed a number of metrics, which include precision, recall (also known as sensitivity or true-positive rate), F1-score, and false-positive rate (FPR) as defined in the below equations.

1 $${\mathrm{Precision}} = \frac{{{\mathrm{TP}}}}{{{\mathrm{TP}}\; +\; {\mathrm{FP}}}}$$

2 $${\mathrm{Recall}} = \frac{{{\mathrm{TP}}}}{{{\mathrm{TP}}\; +\; {\mathrm{FN}}}}$$

3 $${\mathrm{F1}}\,{\mathrm{score}} = 2 \times \frac{{{\mathrm{precision}}\; \times \;{\mathrm{recall}}}}{{{\mathrm{precision}}\; +\; {\mathrm{recall}}}}$$

4 $${\mathrm{FPR}} = \frac{{{\mathrm{FP}}}}{{{\mathrm{FP}}\; +\; {\mathrm{TN}}}}$$

Here, true-positives (TP) are correct seizure predictions, true-negatives (TN) are correct non-seizure predictions, false-positives (FP) are incorrect seizure predictions, and false-negatives (FN) are incorrect non-seizure predictions.

Another metric used is the Area Under the Receiver Operating Characteristic curve (AUROC), where the ROC curve is the recall plotted as a function of the FPR for different cutoff values. Therefore, the AUROC is a more holistic measure of how well a model can distinguish between seizure clips and non-seizure clips regardless of the specific cutoff chosen for classification.

### Network architecture

We use a densely connected inception architecture inspired by Roy et al.^45^ for seizure onset detection. This modeling approach combines the most compelling aspects of deep inception^46^ networks and densely connected network^47^ architectures. Each Inception block, which consists of three convolutional filters with different kernel sizes, is fully connected with other Inception blocks. The model consisted of 8 inception layers followed by two fully connected layers, which resulted in 12,677,803 parameters. Details on the exact model architecture can be found in the Supplementary Methods.

### Model training

Model training for all models is accomplished using the Adam optimizer in PyTorch, with randomly initialized weights. The learning rate is initially set to a value of $10^{ - 6}$ and reduced by a factor of 2 every 10 epochs, and a dropout probability of 0.2 is applied to the last layer. The initial learning rate and dropout probability were found by running a random hyperparameter search and selecting the best values when evaluated on the development set. The plentiful negative examples were under-sampled such that the training set contained 50% positive examples. Batch size was set at 10 EEG signals, the maximum possible (due to memory constraints) for the limiting slow-detection case using the single Titan RTX GPU that was used to train each model. Training each model on the full set of signals for 25 epochs took approximately 12 h for the large weakly labeled dataset on 12-s clips, 44 h on the 60-s clips, and 7 min for the small gold-labeled dataset on 12-s clips, and around 26 min on the 60-s clips.

## Supplementary information

supplementary-materials

Reporting-Summary

## Data Availability

Protected Health Information restrictions apply to the availability of the Stanford clinical datasets presented here, which were used under Institutional Review Board approval for use only in the current study, and so are not publicly available. We hope to make these data available in the future. The Temple University Hospital dataset is publicly available at https://www.isip.piconepress.com/projects/tuh_eeg/html/downloads.shtml. No figures contain associated raw data.

## Code availability

The code used to generate all results in this manuscript can be made available upon request.
