# Supplementary material for: Weak supervision as an efficient approach for automated seizure detection in electroencephalography
Source: NPJ Digit Med. 2020 Apr 20;3:59. doi: 10.1038/s41746-020-0264-0 (PMC7170880; doi:10.1038/s41746-020-0264-0)
Supplement: Supplementary file 1 — supplementary-materials [file 41746_2020_264_MOESM1_ESM.pdf]

Supplementary Notes

Supplementary Note 1: The International League Against Epilepsy (ILAE) defines epileptic seizures as a transient occurrence of signs and/or symptoms due to abnormal excessive or synchronous neuronal activity.[1] In patients who are unable to demonstrate clinical signs or symptoms, such as critically ill or neonatal patients, it is common to extend this definition to include ictal patterns of brain activity without obvious clinical signs/symptoms in the form of electrographic seizures.[1–7] There is some controversy regarding purely electrographic seizures, but from a practical point of view, the presence of electrographic seizures often changes clinical care, and represents an independent predictor of neurological outcomes in a wide variety of clinical settings.[4,7]

Supplementary Note 2: A classification system for seizures and their etiologies including reactive seizures and seizures with epilepsy.

I. Acute Symptomatic Seizures (Reactive Seizures) etiology classification (with additions for febrile seizures and immune etiologies):[7]

   a) Eclampsia
   b) Encephalopathy - include neonatal encephalopathy
   c) Febrile Seizure - added for children
   d) Immune - added from epilepsy category
   e) Infection (CNS Infection)
   f) Metabolic
   g) Neoplastic
   h) Stroke
   i) Toxic
   j) Traumatic
   k) Withdrawal
   l) Other/Unknown

II. Epilepsies:[8]

1. Structural: lesion or malformation present on imaging or pathology, may be acquired due to stroke, HIE, trauma and infection
   a) Ex: hippocampal sclerosis
   b) Gelastic seizures → hypothalamic hamartoma
   c) Rasmussen syndrome may also have a structural component
   d) Polymicrogyria

2. Genetic: includes Genetic Generalized Epilepsies (CAE, JAE, JME, GTC alone)
   a) Single gene disorders: channelopathies, ADNFLE
   b) Childhood absence, JME
   c) Dravet, GEFS+

3. Infectious:
   a) epilepsy results from a core symptom of disorder NOT from acute response to meningitis or encephalitis
   b) Example: neurocysticercosis, TB, HIV, cerebral malaria, SSPE, cerebral toxoplasmosis, Zika virus

c) May also refer to the post infectious development of epilepsy such as viral encephalitis leading to seizures in the aftermath of the acute infection

4. Metabolic:
   a) Results directly from a known or presumed metabolic disorder in which seizures are a core symptom of the disorder
   b) May also have a genetic defect
   c) Examples: porphyria, uremia, aminoacidopathies, pyridoxine-dependent seizures
   d) Acquired: cerebral folate deficiency

5. Immune:
   a) Ex: anti-NMDAR encephalitis
   b) anti-LGI1 encephalitis, limbic encephalitis more generally

6. Unknown: the cause is not yet known

Supplementary Figures

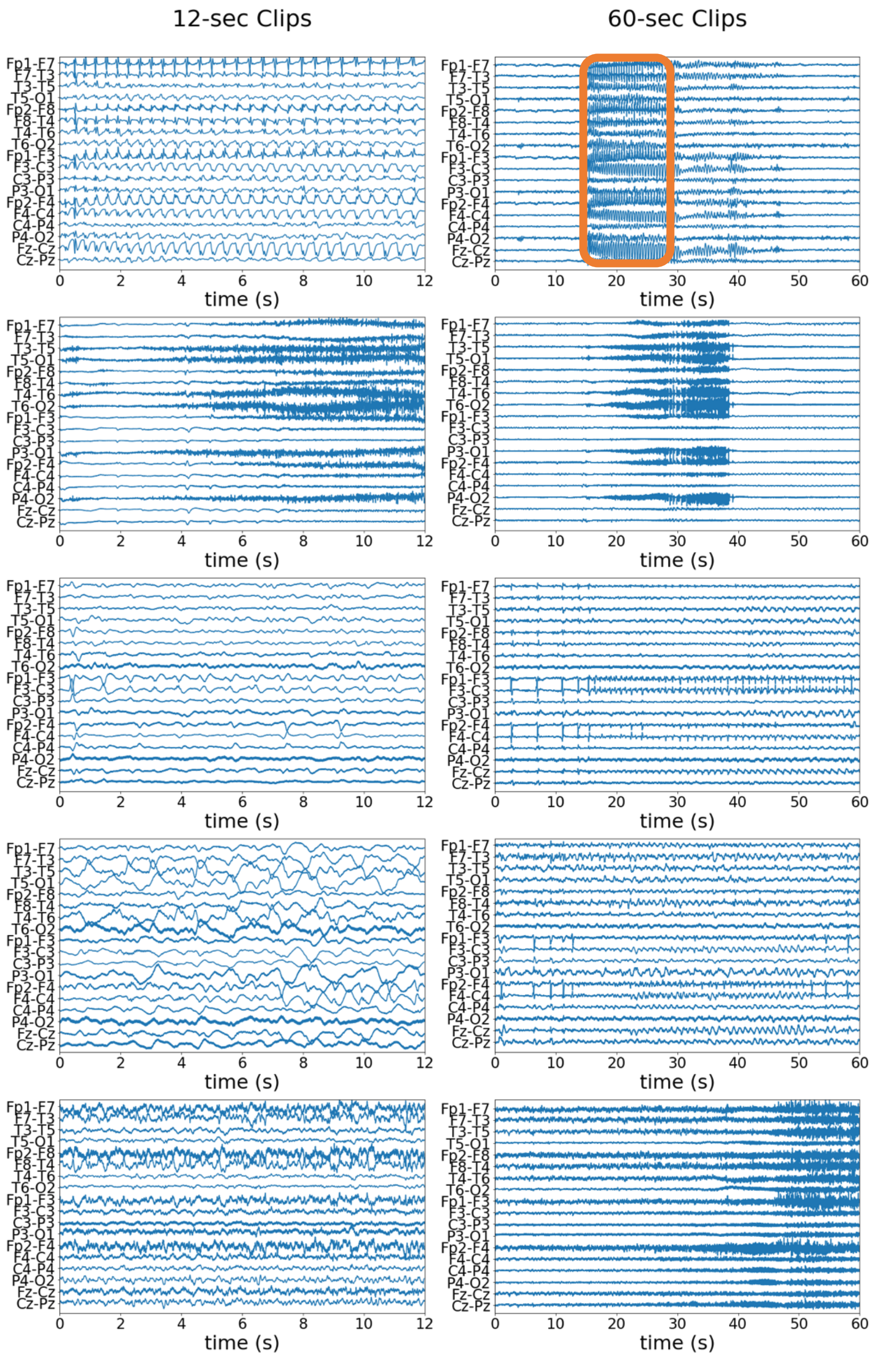

Supplementary Figure 1. Seizure Onset Examples. We chose five random seizure onset cases in our test set and plotted the 12-second and 60-second clips. The 12-second clip corresponds to the time interval [15,27] seconds on the 60-second clip, as shown with the orange rectangle.

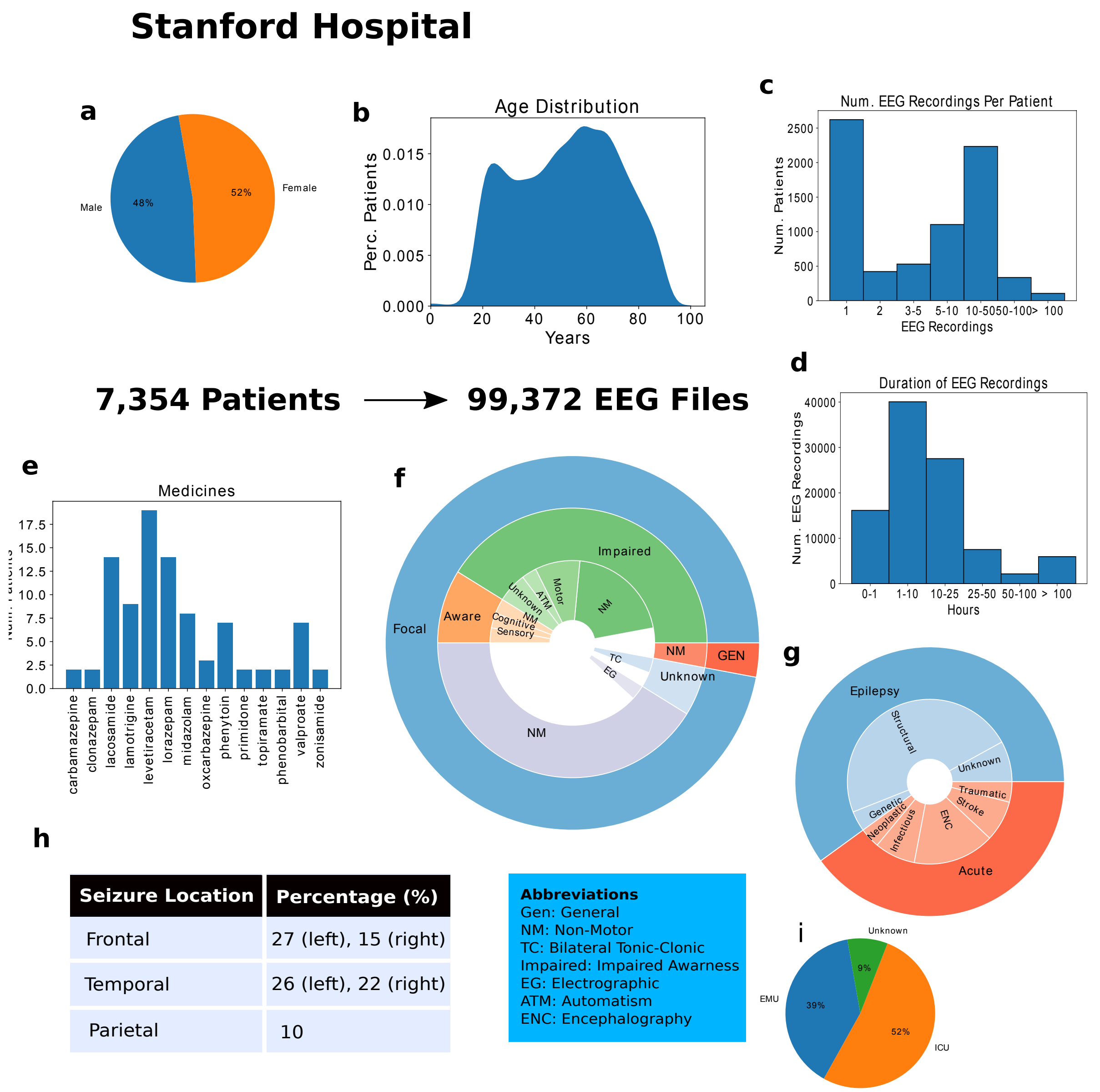

Seizure Location	Percentage (%)
Frontal	27 (left), 15 (right)
Temporal	26 (left), 22 (right)
Parietal	10

Supplementary Figure 2. Data description for Stanford Hospital (a) the gender distribution, (b) the age distribution, (c) the number of EEG recordings per patient, (d) the durations of EEG recordings in hours, (e) a histogram of the medicine types, (f) a hierarchical pie graph describing the different seizure types, (g) a hierarchical pie graph describing etiology types, (h) the percentages of EEG signals collected in different locations, and (i) the distribution of seizure locations. Subfigures (a)-(d) are statistics on the entire dataset, while (e)-(i) are the held-out test set that included 40 seizures.

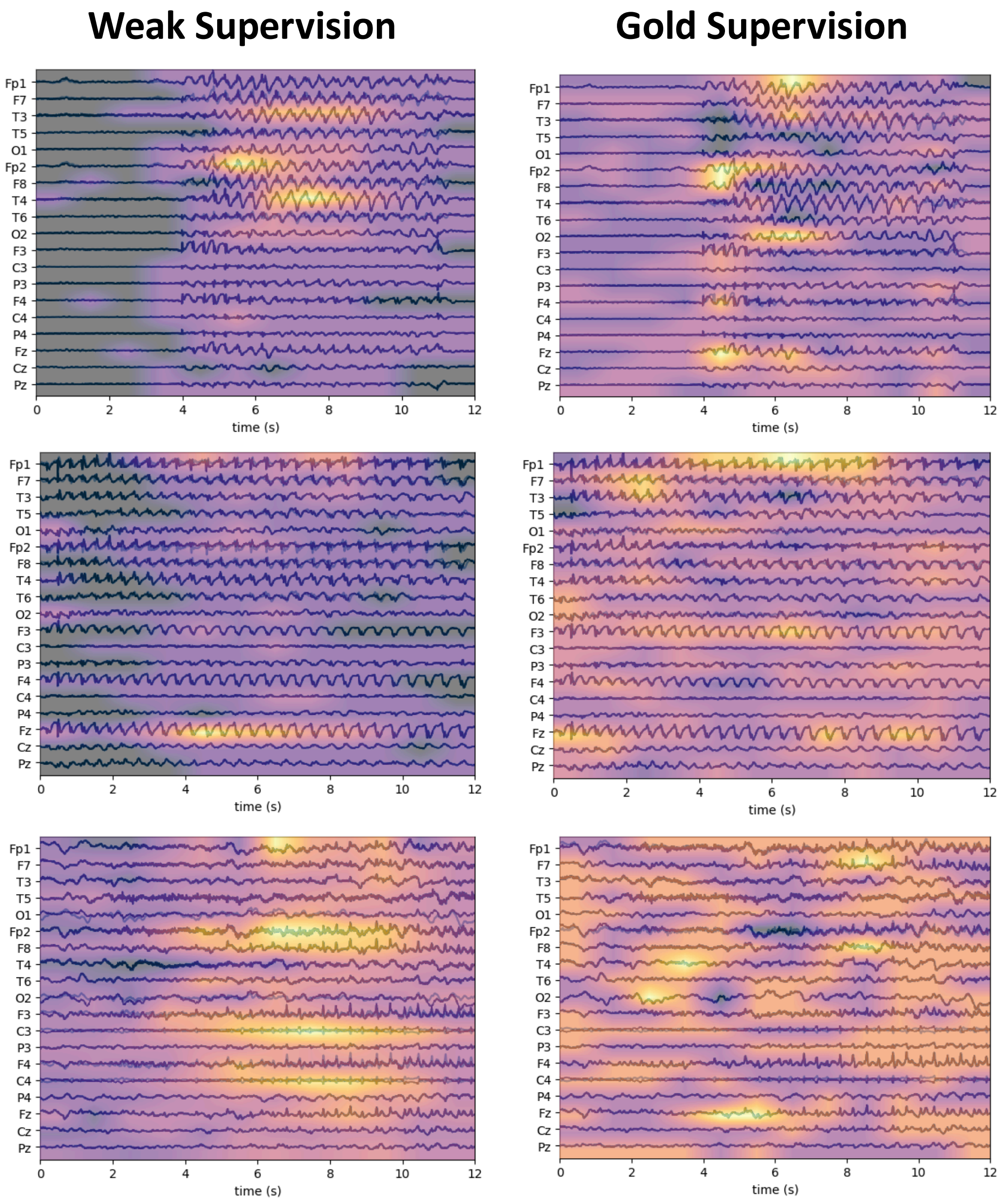

Supplementary Figure 3. Comparing Model Occlusion Maps. Occlusion map comparison on true positive cases between the model trained on small gold-labeled set and the weakly-supervised model.

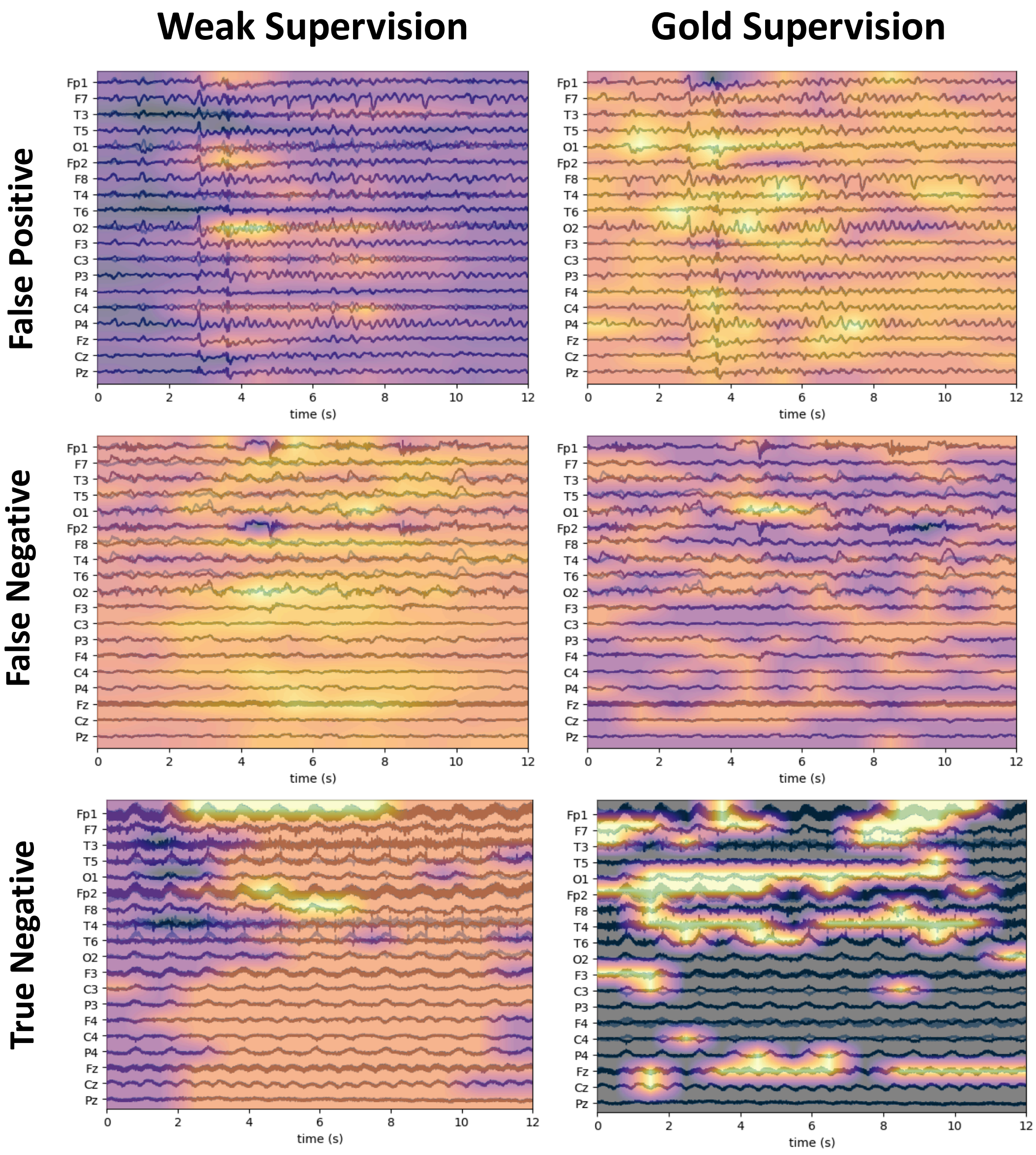

Supplementary Figure 4. Comparing Model Occlusion Maps. Occlusion map comparison on false positive, false negative, and true negative cases between the model trained on small gold-labeled set and the weakly-supervised model.

Supplementary Tables

	Pediatric (F1-score)		Adult (F1-score)	
	12-sec	60-sec	12-sec	60-sec
Logistic Regression	0.25 [0.14,0.40]	0.37 [0.21,0.48]	0.24 [0.13,0.35]	0.38 [0.28, 0.50]
Random Forest	0.38 [0.23,0.54]	0.56 [0.46,0.68]	0.37 [0.24,0.48]	0.58 [0.45, 0.67]
Persyst-13	0.07 [0.0,0.20]	0.61 [0.38,0.69]	0 [0,0]	0.65 [0.44,0.80]
Dense-CNN (small gold-standard set)	0.29 [0.16,0.40]	0.60 [0.44,0.72]	0.28 [0.15,0.36]	0.41 [0.22,0.56]
Dense-CNN (weak annotations)	0.67 [0.51,0.77]	0.77 [0.60,0.84]	0.49 [0.27,0.64]	0.76 [0.55,0.87]

Supplementary Table 1. Comparison of baseline models to the CNN model using the F1-score with 95% confidence intervals. The confidence intervals were calculated using 1,000 bootstrap samples from the test set with sample size 60% of the original test set size. For the Dense-CNN, median models were used to compute confidence intervals. Computed p-values (two-sided t-test) comparing the mean F1-score of the Dense-CNN on weak annotations to each baseline are all less than 0.05 indicating that the F1-score gains are statistically meaningful.

	Pediatric (FPR)		Adult (FPR)	
	12-sec	60-sec	12-sec	60-sec
Logistic Regression	0.36 [0.29,0.46]	0.50 [0.41,0.64]	0.56 [0.48,0.65]	0.55 [0.47, 0.65]
Random Forest	0.19 [0.13,0.27]	0.52 [0.45,0.67]	0.61 [0.53,0.70]	0.52 [0.44,61]
Persyst-13	0.015 [0,0.04]	0.10 [0.05,0.17]	0 [0,0.02]	0.054 [0.01,0.10]
Dense-CNN (gold-small)	0.39 [0.30,0.48]	0.20 [0.12,0.25]	0.47 [0.39,0.55]	0.16 [0.09,0.21]
Dense-CNN (weak-large)	0.13 [0.09,0.22]	0.10 [0.06,0.16]	0.14 [0.08,0.20]	0.079 [0.02,0.10]

Supplementary Table 2. Comparison of baseline models to the CNN model using the FPR with 95% confidence intervals. The confidence intervals were calculated using 1,000 bootstrap samples from the test set with sample size 60% of the original test set size. For the Dense-CNN, median models were used to compute confidence intervals. Computed p-values (two-sided t-test) comparing the mean FPR of the Dense-CNN on weak annotations to each baseline are all less than 0.05 indicating that the FPR gains are statistically meaningful, with the exception of Persyst-13 over 60-sec clips.

Supplementary Methods

The Dense-Inception Architecture

We have divided the different parts of our dense-inception layer into the following blocks and provided the detailed architectural parameters.

```
Head Convolutional Layers:

        (branchA_1): BasicConv2d(
          (conv): Conv2d(1, 10, kernel_size=(9, 1), stride=(1, 1), padding=(4, 0))
        )
        (branchA_2): BasicConv2d(
          (conv): Conv2d(10, 10, kernel_size=(9, 1), stride=(1, 1), padding=(4, 0))
        )
        (branchB_1): BasicConv2d(
          (conv): Conv2d(1, 10, kernel_size=(15, 1), stride=(1, 1), padding=(7, 0))
        )
        (branchB_2): BasicConv2d(
          (conv): Conv2d(10, 10, kernel_size=(15, 1), stride=(1, 1), padding=(7, 0))
        )
        (branchB_3): BasicConv2d(
          (conv): Conv2d(10, 10, kernel_size=(15, 1), stride=(1, 1), padding=(7, 0))
        )
        (branchC_1): BasicConv2d(
          (conv): Conv2d(1, 10, kernel_size=(21, 1), stride=(1, 1), padding=(10, 0))
        )
        (branchC_2): BasicConv2d(
          (conv): Conv2d(10, 10, kernel_size=(21, 1), stride=(1, 1), padding=(10, 0))
        )
      )
```

```
Inception Layer #1:

      (inception_1): Inception4(
        (branchA_1): BasicConv2d(
          (conv): Conv2d(30, 30, kernel_size=(9, 1), stride=(1, 1), padding=(4, 0))
        )
        (branchA_2): BasicConv2d(
          (conv): Conv2d(30, 30, kernel_size=(9, 1), stride=(1, 1), padding=(4, 0))
        )
        (branchB_1): BasicConv2d(
          (conv): Conv2d(30, 30, kernel_size=(13, 1), stride=(1, 1), padding=(6, 0))
        )
        (branchB_2): BasicConv2d(
          (conv): Conv2d(30, 30, kernel_size=(13, 1), stride=(1, 1), padding=(6, 0))
        )
        (branchB_3): BasicConv2d(
          (conv): Conv2d(30, 30, kernel_size=(13, 1), stride=(1, 1), padding=(6, 0))
        )
        (branchC_1): BasicConv2d(
          (conv): Conv2d(30, 30, kernel_size=(21, 1), stride=(1, 1), padding=(10, 0))
        )
        (branchC_2): BasicConv2d(
          (conv): Conv2d(30, 30, kernel_size=(17, 1), stride=(1, 1), padding=(8, 0))
        )
      )
      (conv1x1_10): BasicConv2d(
        (conv): Conv2d(120, 90, kernel_size=(1, 1), stride=(1, 1))
      )
```

Inception Layer #2:

```
(inception_2): Inception4(
  (branchA_1): BasicConv2d(
    (conv): Conv2d(90, 90, kernel_size=(7, 1), stride=(1, 1), padding=(3, 0))
  )
  (branchA_2): BasicConv2d(
    (conv): Conv2d(90, 90, kernel_size=(7, 1), stride=(1, 1), padding=(3, 0))
  )
  (branchB_1): BasicConv2d(
    (conv): Conv2d(90, 90, kernel_size=(11, 1), stride=(1, 1), padding=(5, 0))
  )
  (branchB_2): BasicConv2d(
    (conv): Conv2d(90, 90, kernel_size=(11, 1), stride=(1, 1), padding=(5, 0))
  )
  (branchB_3): BasicConv2d(
    (conv): Conv2d(90, 90, kernel_size=(11, 1), stride=(1, 1), padding=(5, 0))
  )
  (branchC_1): BasicConv2d(
    (conv): Conv2d(90, 90, kernel_size=(21, 1), stride=(1, 1), padding=(10, 0))
  )
  (branchC_2): BasicConv2d(
    (conv): Conv2d(90, 90, kernel_size=(15, 1), stride=(1, 1), padding=(7, 0))
  )
)
(conv1x1_2): BasicConv2d(
  (conv): Conv2d(270, 180, kernel_size=(1, 1), stride=(1, 1))
)
```

Inception Layer #3:

```
(inception_3): Inception4(
  (branchA_1): BasicConv2d(
    (conv): Conv2d(180, 180, kernel_size=(5, 1), stride=(1, 1), padding=(2, 0))
  )
  (branchA_2): BasicConv2d(
    (conv): Conv2d(180, 180, kernel_size=(5, 1), stride=(1, 1), padding=(2, 0))
  )
  (branchB_1): BasicConv2d(
    (conv): Conv2d(180, 180, kernel_size=(7, 1), stride=(1, 1), padding=(3, 0))
  )
  (branchB_2): BasicConv2d(
    (conv): Conv2d(180, 180, kernel_size=(7, 1), stride=(1, 1), padding=(3, 0))
  )
  (branchB_3): BasicConv2d(
    (conv): Conv2d(180, 180, kernel_size=(7, 1), stride=(1, 1), padding=(3, 0))
  )
  (branchC_1): BasicConv2d(
    (conv): Conv2d(180, 180, kernel_size=(21, 1), stride=(1, 1), padding=(10, 0))
  )
  (branchC_2): BasicConv2d(
    (conv): Conv2d(180, 180, kernel_size=(9, 1), stride=(1, 1), padding=(4, 0))
  )
)
(conv1x1_3): BasicConv2d(
  (conv): Conv2d(540, 180, kernel_size=(1, 1), stride=(1, 1))
)
(conv1x1_32): BasicConv2d(
  (conv): Conv2d(360, 180, kernel_size=(1, 1), stride=(1, 1))
)
```

Inception Layer #4:

```
(inception_4): Inception4(
        (branchA_1): BasicConv2d(
          (conv): Conv2d(180, 180, kernel_size=(3, 1), stride=(1, 1), padding=(1, 0))
        )
        (branchA_2): BasicConv2d(
          (conv): Conv2d(180, 180, kernel_size=(3, 1), stride=(1, 1), padding=(1, 0))
        )
        (branchB_1): BasicConv2d(
          (conv): Conv2d(180, 180, kernel_size=(5, 1), stride=(1, 1), padding=(2, 0))
        )
        (branchB_2): BasicConv2d(
          (conv): Conv2d(180, 180, kernel_size=(5, 1), stride=(1, 1), padding=(2, 0))
        )
        (branchB_3): BasicConv2d(
          (conv): Conv2d(180, 180, kernel_size=(5, 1), stride=(1, 1), padding=(2, 0))
        )
        (branchC_1): BasicConv2d(
          (conv): Conv2d(180, 180, kernel_size=(21, 1), stride=(1, 1), padding=(10, 0))
        )
        (branchC_2): BasicConv2d(
          (conv): Conv2d(180, 180, kernel_size=(7, 1), stride=(1, 1), padding=(3, 0))
        )
      )
      (conv1x1_4): BasicConv2d(
        (conv): Conv2d(540, 180, kernel_size=(1, 1), stride=(1, 1))
      )
```

Inception Layer #5:

```
      (inception_5): Inception4(
        (branchA_1): BasicConv2d(
          (conv): Conv2d(180, 180, kernel_size=(3, 1), stride=(1, 1), padding=(1, 0))
        )
        (branchA_2): BasicConv2d(
          (conv): Conv2d(180, 180, kernel_size=(3, 1), stride=(1, 1), padding=(1, 0))
        )
        (branchB_1): BasicConv2d(
          (conv): Conv2d(180, 180, kernel_size=(5, 1), stride=(1, 1), padding=(2, 0))
        )
        (branchB_2): BasicConv2d(
          (conv): Conv2d(180, 180, kernel_size=(5, 1), stride=(1, 1), padding=(2, 0))
        )
        (branchB_3): BasicConv2d(
          (conv): Conv2d(180, 180, kernel_size=(5, 1), stride=(1, 1), padding=(2, 0))
        )
        (branchC_1): BasicConv2d(
          (conv): Conv2d(180, 180, kernel_size=(21, 1), stride=(1, 1), padding=(10, 0))
        )
        (branchC_2): BasicConv2d(
          (conv): Conv2d(180, 180, kernel_size=(7, 1), stride=(1, 1), padding=(3, 0))
        )
      )
      (conv1x1_5): BasicConv2d(
        (conv): Conv2d(540, 270, kernel_size=(1, 1), stride=(1, 1))
      )
      (conv1x1_54): BasicConv2d(
        (conv): Conv2d(450, 180, kernel_size=(1, 1), stride=(1, 1))
      )
```

Inception Layer #6:

```
      (inception_6): Inception4(
        (branchA_1): BasicConv2d(
          (conv): Conv2d(180, 180, kernel_size=(3, 1), stride=(1, 1), padding=(1, 0))
        )
        (branchA_2): BasicConv2d(
          (conv): Conv2d(180, 180, kernel_size=(3, 1), stride=(1, 1), padding=(1, 0))
        )
        (branchB_1): BasicConv2d(
          (conv): Conv2d(180, 180, kernel_size=(5, 1), stride=(1, 1), padding=(2, 0))
        )
        (branchB_2): BasicConv2d(
          (conv): Conv2d(180, 180, kernel_size=(5, 1), stride=(1, 1), padding=(2, 0))
        )
        (branchB_3): BasicConv2d(
          (conv): Conv2d(180, 180, kernel_size=(5, 1), stride=(1, 1), padding=(2, 0))
        )
        (branchC_1): BasicConv2d(
          (conv): Conv2d(180, 180, kernel_size=(21, 1), stride=(1, 1), padding=(10, 0))
        )
        (branchC_2): BasicConv2d(
          (conv): Conv2d(180, 180, kernel_size=(7, 1), stride=(1, 1), padding=(3, 0))
        )
      )
      (conv1x1_6): BasicConv2d(
        (conv): Conv2d(540, 180, kernel_size=(1, 1), stride=(1, 1))
      )
```

Inception Layer #7:

```
(inception_7): Inception4(
        (branchA_1): BasicConv2d(
          (conv): Conv2d(180, 180, kernel_size=(3, 1), stride=(1, 1), padding=(1, 0))
        )
        (branchA_2): BasicConv2d(
          (conv): Conv2d(180, 180, kernel_size=(3, 1), stride=(1, 1), padding=(1, 0))
        )
        (branchB_1): BasicConv2d(
          (conv): Conv2d(180, 180, kernel_size=(5, 1), stride=(1, 1), padding=(2, 0))
        )
        (branchB_2): BasicConv2d(
          (conv): Conv2d(180, 180, kernel_size=(5, 1), stride=(1, 1), padding=(2, 0))
        )
        (branchB_3): BasicConv2d(
          (conv): Conv2d(180, 180, kernel_size=(5, 1), stride=(1, 1), padding=(2, 0))
        )
        (branchC_1): BasicConv2d(
          (conv): Conv2d(180, 180, kernel_size=(21, 1), stride=(1, 1), padding=(10, 0))
        )
        (branchC_2): BasicConv2d(
          (conv): Conv2d(180, 180, kernel_size=(7, 1), stride=(1, 1), padding=(3, 0))
        )
      )
      (conv1x1_7): BasicConv2d(
        (conv): Conv2d(540, 270, kernel_size=(1, 1), stride=(1, 1))
      )
      (conv1x1_76): BasicConv2d(
        (conv): Conv2d(450, 360, kernel_size=(1, 1), stride=(1, 1))
      )
```

Classifier:

```
(fc1): Linear(in_features=20520, out_features=128, bias=True)
      (fcbn1): BatchNorm1d(128, eps=1e-05, momentum=0.1, affine=True, track_running_stats=True)
      (fc2): Linear(in_features=128, out_features=1, bias=True)
```

Supplementary References

1. Fisher, R. S. *et al.* ILAE Official Report: A practical clinical definition of epilepsy. *Epilepsia* (2014). doi:10.1111/epi.12550
2. Brandon Westover, M. *et al.* The probability of seizures during EEG monitoring in critically ill adults. *Clin. Neurophysiol.* **126**, 463–471 (2015).
3. Williams, K., Jarrar, R. & Buchhalter, J. Continuous video-EEG monitoring in pediatric intensive care units. *Epilepsia* **52**, 1130–1136 (2011).
4. Abend, N. S., Wusthoff, C. J., Goldberg, E. M. & Dlugos, D. J. Electrographic seizures and status epilepticus in critically ill children and neonates with encephalopathy. *The Lancet Neurology* (2013). doi:10.1016/S1474-4422(13)70246-1
5. Worden, L. T. *et al.* The probability of seizures during continuous EEG monitoring in high-risk neonates. *Epilepsia* epi.16387 (2019). doi:10.1111/epi.16387
6. Megiddo, I. *et al.* Health and economic benefits of public financing of epilepsy treatment in India: An agent-based simulation model. *Epilepsia* (2016). doi:10.1111/epi.13294
7. Ott, D. *et al.* Behavioral disorders in pediatric epilepsy: unmet psychiatric need. *Epilepsia* (2003). doi:10.1046/j.1528-1157.2003.25002.x
8. Kerr, M. P. The impact of epilepsy on patients' lives. *Acta Neurologica Scandinavica* (2012). doi:10.1111/ane.12014
